# Priming with biocides: A pathway to antibiotic resistance?

**DOI:** 10.1111/jam.15564

**Published:** 2022-05-16

**Authors:** Pat Adkin, Andrew Hitchcock, Laura J. Smith, Susannah E. Walsh

**Affiliations:** ^1^ Leicester School of Pharmacy Hawthorn Building, De Montfort University Leicester UK; ^2^ School of Biosciences University of Sheffield Sheffield UK; ^3^ School of Pharmacy and Life Sciences Robert Gordon University Aberdeen UK

## Abstract

**Aims:**

To investigate the priming effects of sub‐inhibitory concentrations of biocides on antibiotic resistance in bacteria.

**Methods and results:**

*Escherichia coli*, *Pseudomonas aeruginosa* and *Staphylococcus aureus* were exposed to sub‐inhibitory concentrations of biocides via a gradient plate method. Minimum inhibitory concentration (MIC) and antibiotic susceptibility were determined, and efflux pump inhibitors (thioridazine and chlorpromazine) were used to investigate antibiotic resistance mechanism(s). *Escherichia coli* displayed a twofold increase in MIC (32–64 mg l^−1^) to H_2_O_2_ which was stable after 15 passages, but lost after 6 weeks, and *P*. *aeruginosa* displayed a twofold increase in MIC (64–128 mg l^−1^) to BZK which was also stable for 15 passages. There were no other tolerances observed to biocides in *E*. *coli*, *P*. *aeruginosa* or *S*. *aureus*; however, stable cross‐resistance to antibiotics was observed in the absence of a stable increased tolerance to biocides. Sixfold increases in MIC to cephalothin and fourfold to ceftriaxone and ampicillin were observed in hydrogen peroxide primed *E. coli*. Chlorhexidine primed *S. aureus* showed a fourfold increase in MIC to oxacillin, and glutaraldehyde‐primed *P. aeruginosa* showed fourfold (sulphatriad) and eightfold (ciprofloxacin) increases in MIC. Thioridazine increased the susceptibility of *E. coli* to cephalothin and cefoxitin by fourfold and twofold, respectively, and both thioridazine and chlorpromazine increased the susceptibility *S. aureus* to oxacillin by eightfold and fourfold, respectively.

**Conclusions:**

These findings demonstrate that sub‐inhibitory concentrations of biocides can prime bacteria to become resistant to antibiotics even in the absence of stable biocide tolerance and suggests activation of efflux mechanisms may be a contributory factor.

**Significance and Impact of the Study:**

This study demonstrates the effects of low‐level exposure of biocides (priming) on antibiotic resistance even in the absence of obvious increased biocidal tolerance.

## INTRODUCTION

The rise in multidrug‐resistant bacteria means that some conventional antimicrobials have become ineffective in the treatment of infections (Roca Subirà et al., 2012; Tuon et al., 2012; Walsh & Toleman, 2011). Biocides have been used for centuries to control infectious agents (Maillard, 2002; Morente et al., 2013; Russell, 2002). They are either applied as or added to formulated products that are used as disinfectants, preservatives, pesticides, antiseptics and even cosmetics (Gilbert & McBain, 2003; Knapp et al., 2015; Maillard, 2002). Public awareness to infection control has caused a rise in the use of biocide and biocidal products in the home environment. As a result of increased use of biocides, especially at sub‐inhibitory concentrations, there are concerns over how their selective pressure might potentially favour the development of less susceptible bacterial strains, as well as encourage the expression and dissemination of resistance mechanisms to biocides and other antimicrobial agents (Fraise, 2002; Knapp et al., 2015; Maillard et al., 2013; McBain & Gilbert, 2001; Pereira et al., 2021; SCENIHR, 2010). There are several laboratory studies demonstrating a link between exposure of bacteria to sub‐inhibitory concentrations and increased tolerance and resistance to biocides (Bock et al., 2016; Christensen et al., 2011; Escalada et al., 2005; Knapp et al., 2013; Walsh et al., 2003). Some studies show both increased tolerance to biocides plus antibiotic cross‐resistance in bacteria after exposure to sub‐inhibitory concentrations of biocides (Kurenbach et al., 2015; Wand et al., 2016). While exposure to biocides has been linked to reduced susceptibility and cross‐resistance to antibiotics (Braoudaki & Hilton, 2004; Chuanchuen et al., 2001; Slayden et al., 2000; Soumet et al., 2012; Tkachenko et al., 2007; Wand, 2017), it is paramount that we understand what happens in the absence of obvious increased biocidal tolerance after low‐level exposure; will there be a change in bacterial resistance to antibiotics in that instance?

Since biocides have several target sites within bacteria (Maillard, 2002; Russell, 2002), the most common mechanisms for cross‐resistance are via non‐specific processes such as efflux‐pumps (Bogomolnaya et al., 2013; Costa et al., 2013) and changes in properties of their cell wall, for example reduced permeability due to porin downregulation (Jaffe et al., 1982; Manzoor et al., 1999).

A review by Maillard et al. (2013) and a report from the scientific committee for emerging and newly identified health risks (SCENIHR, 2010) identified biocides as a risk due to selective pressure for less susceptible strains. Their findings highlighted a key gap in current knowledge in understanding the effect of low concentrations of biocides on bacterial cells, as well as the mechanisms involved in the development of resistance and cross‐resistance (Jaffe et al., 1982; Knapp et al., 2015; Maillard, 2002; SCENIHR, 2010). Because of these concerns, the European Union and the United States have proposed regulatory changes requiring manufacturers of biocidal products to provide data on the risks of resistance development in organisms targeted by biocidal products (Knapp et al., 2015; SCENIHR, 2010).

As previously demonstrated by several studies, prolonged exposure of bacteria to biocides under laboratory conditions can generate less susceptible mutants which can often display reduced susceptibility to various antibiotics (Fernández Márquez et al., 2017; Hardy et al., 2017; Karatzas et al., 2007, 2008; Randall et al., 2007; Whitehead et al., 2011).

The present study investigates the effect of biocide priming on bacterial resistance to antibiotics after continuous exposure of *Escherichia coli*, *Staphylococcus aureus* and *Pseudomonas aeruginosa* to low concentrations of hydrogen peroxide (H_2_O_2_), chlorhexidine (CHG), benzalkonium chloride (BZK) and glutaraldehyde (GTA). The three organisms used are all commonly associated with hospital‐acquired infections (HAIs) and the strains selected are those listed in the BS EN 1276:2009 disinfection test method. *Escherichia coli* is a Gram‐negative bacteria commonly transmitted to humans via the consumption of contaminated food and water (Kaper et al., 2004) and known to cause infections such as diarrhoea and haemorrhagic colitis to severe complications such as haemolytic anaemia and acute renal failure (Brzuszkiewicz et al., 2011). Outer membrane protein modification like the downregulation of porins and upregulation of efflux pumps such as AcrAB have been linked with increased antimicrobial resistance in *E. coli* (Ma et al., 1993, 1994). *Staphylococcus aureus* is a Gram‐positive, opportunistic pathogen responsible for both hospital‐ and community‐acquired infections (Boucher et al., 2010; Latimer et al., 2012). Upregulation of QacA‐D, E and H (Heir et al., 1998; Kazama et al., 1998; Rouch et al., 1990) and NorA, NorB and NorC efflux pumps (Truong‐Bolduc et al., 2006) have been linked to antimicrobial resistance in *S. aureus*. *Pseudomonas aeruginosa* is an opportunistic, intrinsically resistant, Gram‐negative bacteria that is responsible for a range of infections to the eyes and ears to serious complications in cystic fibrosis and bronchiectasis patients (Lambert, 2002; Soothill, 2013). Upregulation of efflux pumps such as MexAB, MexCD and MexEF and outer membrane protein modification have been linked to antimicrobial resistance in *P. aeruginosa* (Poole, 2001; Schweizer, 1998). This study aims to understand the impact of prolonged exposure of bacteria to low concentrations of biocide, and the possible cross‐resistance to antibiotics.

## MATERIALS AND METHODS

### Bacterial strains and storage

*Escherichia coli* ATCC 8739, *P. aeruginosa* ATCC 15442 and *S. aureus* ATCC 6538 strains were grown in tryptone soya broth (TSB) (Oxoid) at 37°C with shaking at 100 rev min^−1^ for 24 h and stored on protect beads (Scientific Laboratory Supplies Limited) at −80°C.

### Preparation of inoculum

Bacterial strains were grown on Mueller Hinton agar (MHA) (Oxoid) at 37°C for 24 h and a single colony was transferred to 10 ml sterile saline. The turbidity of the suspension was adjusted to the equivalence 0.5 McFarland (absorbance range between 0.08 and 0.13) at 625 nm line with the European Committee for Antimicrobial Susceptibility Testing (EUCAST, 2015) and of the European Society of Clinical Microbiology and Infectious Diseases [ESCMID], 2000).

### Preparation of biocide working solutions

A twofold dilution series of biocides (mg l^−1^) was prepared in Mueller‐Hinton broth following the BS EN ISO 20776‐6 guidelines/protocol (BSI, 2006). Hydrogen peroxide (H_2_O_2_) concentration range 1–512 mg l^−1^ (Fisher Scientific Belgium), formulated chlorhexidine gluconate (CHG) concentration range 0.976–500 mg l^−1^ (Molnlycke Health Care Ltd), benzalkonium chloride (BZK) concentration range 1–512 mg l^−1^ and glutaraldehyde (GTA) concentration range 8–4096 mg l^−1^ (Sigma‐Aldrich).

### Preparation of antibiotic working solutions

Antibiotic discs Mastring‐S (M13 and M14) were obtained from Mast Diagnostics UK. Antibiotics and efflux pump inhibitors (EPIs) were purchased from Sigma Aldrich and used at the following concentrations: oxacillin sodium salt concentration 0.0020–8 mg l^−1^, cefoxitin sodium salt 0.25–32 mg l^−1^, cephalothin sodium salt 0.25–32 mg l^−1^, ceftriaxone disodium salt hemi (heptahydrate) 0.016–2 mg l^−1^, ampicillin 0.25–32 mg l^−1^, sulfathiazole 37% v/v, sulfadiazine 37% v/v, sulfamerazine 26% v/v, ciprofloxacin sodium salt 0.125–2 mg l^−1^, thioridazine hydrochloride (TZ) 1.9–500 mg l^−1^ and chlorpromazine hydrochloride (CPZ) 4–256 mg l^−1^. Stock solutions were prepared by dissolving salts in sterile distilled water and working solutions were prepared in Mueller‐Hinton broth following the BS EN ISO 20776‐6 guidelines/protocol (BSI, 2006).

### Adaptation of bacterial strains to biocides using gradient plate

A twofold dilution series of biocides were prepared in sterile distilled water following the EUCAST guidelines of ESCMID (EUCAST, 2015; ESCMID 2000). The required biocide (1 ml) was added to 19 ml sterile molten nutrient agar to give specific final concentrations (from a concentration just below the MIC of the biocide against tested bacteria strain). The molten agar and biocide mixture was poured into sterile Petri dishes and set at an angle, after which plates were placed on a flat surface and 20 ml sterile molten agar were poured over the first layer and allowed to set. Plates were left at 4°C for 24 h to allow diffusion of biocide. The 24 h bacterial cultures were streaked along the concentration gradient starting at the point of the gradient plate containing the lowest concentration of biocide. These streaked plates were incubated at 37°C for 24 h. Bacterial colonies that grew the furthest along the gradient towards the high concentration were used to streak a new gradient plate containing the next highest concentration, until no further increases in tolerance were observed, at which point the furthest‐growing colonies were selected and stored on protect beads at −80°C. For example, the H_2_O_2_ MIC against *E. coli* was 32 mg l^−1^; therefore, the bacteria was first streaked on a plate containing 16 mg l^−1^ H_2_O_2_ (concentration twofold below the MIC), then moved to 32, 64, 128 and 256 mg l^−1^ until no growth were observed on the 512 mg l^−1^ plate.

### Stability of adaptive resistance

To confirm the stability of the primed strains produced, the gradient plate isolates were subcultured 15 times in 10 ml of TSB without biocide. MIC between parent and primed strains were compared after 1, 2, 10 and 15 subcultures, following EUCAST guidelines ESCMID (EUCAST, 2015; ESCMID 2000).

### Determination of minimum inhibitory concentrations

The minimum inhibitory concentrations (MICs) were determined for each strain before and after exposure to the biocides. This was carried out using the colony suspension method of testing in accordance with BS EN ISO 20776‐1 guidelines/protocol (BSI, 2006). Briefly, 50 μl of the standardized inoculum was dispensed into each well of a 96‐well plate containing 50 μl of the appropriate concentration of biocides or antimicrobial agents to give a final inoculum concentration of 5 × 10^5^ CFU per ml. The inoculated 96‐well plate was incubated at 37°C for 24 h. The lowest concentration of compound that prevented bacterial growth after 24 h of incubation was used to establish the MIC. Bacterial growth was determined in two ways: by visual observation of growth on the 96‐well plate and by measuring absorbance at 625 nm in a Spectramax plus plate reader. All experiments were carried out as biological and technical triplicates.

### Antibiotic susceptibility testing using disc diffusion test

The antibiotic susceptibility of each bacterial strain was determined before and after biocide treatment with biocides using the disc diffusion method of antimicrobial susceptibility testing. This was done in accordance with the guidelines of European committee on antimicrobial susceptibility testing (EUCAST, 2015). Briefly, antibiotic discs were applied to surfaces of the inoculated plates within 15 min of inoculation. After 24 h of incubation at 37°C, zones of inhibition were measured with a digital Vernier calliper.

### The effect of EPIs

Standardized bacterial suspensions of primed strains were prepared from 24 h culture as previously described. Suspensions were grown in 96‐well plates for 24 h at 37°C in concentrations of antibiotics they were resistant to, plus an EPI at half its MIC (a concentration that does not inhibit growth of strain). H_2_O_2_ primed *E. coli* (EcH_2_O), GTA primed *P. aeruginosa* (PaGTA) and BZK primed *P. aeruginosa* (PaBZK) were tested with TZ, and CHG primed *S. aureus* (SaCHG) was tested with CPZ and TZ. EcH_2_O, 40 mg l^−1^ TZ was tested with cephalothin and cefoxitin, PaGTA, 250 mg l^−1^ TZ was tested with ciprofloxacin and sulphatriad, PaBZK 250 mg l^−1^ TZ was tested with BZK, and SaCHG was tested with oxacillin with either 32 mg l^−1^ CPZ or 15.625 mg l^−1^ TZ. The lowest concentration of antibiotic plus EPI that prevented bacterial growth after 24 h of incubation was used to establish MIC.

### Confirmation of strain identity

16S rRNA genes were amplified from genomic DNA of parent and biocide‐treated strains using Q5® Hot Start High‐Fidelity 2X Master Mix (New England Biolabs) and primer pairs 16S rRNA For 1 (5′‐AGAGTTTGATCCTGGCTCAG‐3′) and 16S rRNA Rev 1 (5′‐ACGGCTACCTTGTTACGACTT‐3′) for *S. aureus*, 16S rRNA For 2 (5′‐AGAGTTTGATCATGGCTCAG‐3′) and 16S rRNA Rev 2 (5′‐ACGGTTACCTTGTTACGACTT‐3′) for *E. coli*, and 16S rRNA For 2 and 16S rRNA Rev 1 for *P. aeruginosa*. PCR products were analysed on 0.8% (w/v) agarose gels and sequenced by automated DNA sequencing (Eurofins).

### Fitness of primed strains

Overnight cultures of parent and primed bacterial strains were inoculated in antibiotic‐free Mueller Hinton broth (MHB) and absorbance at 600 nm was recorded every 10 min for 16 h. Resulting data and standard deviations were plotted. Each experiment was conducted in biological triplicates.

## RESULTS

### Effect of sub‐inhibitory concentrations of biocides

The MIC of four biocides against pre‐ and post‐primed strains of bacteria are summarized in Table 1. There was an initial increase in MIC to H_2_O_2_ from 32 mg l^−1^ for the *E. coli* parent strain to 64 mg l^−1^ after H_2_O_2_ priming (EcH_2_O_2_), which was stable after 15 passages but lost after storage for 6 weeks. The MIC of *E. coli* did not change after exposure to other biocides used in this study. Apart from a twofold increased MIC from 64 mg l^−1^ for the parent strain of *P. aeruginosa* to 128 mg l^−1^ (stable after 15 passages) after BZK priming (PaBZK), there was no other obvious increased tolerance observed to biocides tested in *P. aeruginosa*. No biocide tolerance was observed with *S. aureus*, but bacteria exposed to CHG (SaCHG) were included in further testing to determine whether there was any observed antibiotic cross‐resistance.

**TABLE 1:** MIC of biocides against pre‐ and post‐primed strains of bacteria

Strain	MIC (mg l^−1^), *n* = 3			
	Hydrogen peroxide	Glutaraldehyde	Benzalkonium chloride	Chlorhexidine
*E. coli*	32	1024	16	15.6
EcH_2_O_2_	64^a^	1024	16	15.6
*S. aureus*	4	512	4	7.8
SaCHG	4	512	4	7.8
*P. aeruginosa*	32	1024	64	31.3
PaGTA	32	1024	—	31.3
PaBZK	—	—	128^b^	—

*Note*: EcH_2_O_2,_ H_2_O_2_ primed *E. coli*; SaCHG, CHG primed *S. aureus*; PaGTA, GTA primed *P. aeruginosa;* and PaBZK, BZK primed *P. aeruginosa*.

^a^ EcH_2_O_2_ tolerance to H_2_O_2_ was unstable.

^b^ Only in PaBZK was there a stable twofold increase to tolerance BZK.

The results of antibiotic susceptibility tests for parent and primed strains of bacteria are summarized in Table 2. Two sample *t*‐tests assuming unequal variances (*p* = 0.05) were used to compared the data and statistically significant changes were observed in the following five cases. Changes in zones of inhibition were observed in EcH_2_O_2_, where there was no zone of inhibition around cephalothin or ampicillin discs compared to the parent strain *E. coli* strain, where 10.43 mm (cephalothin) and 18.88 mm (ampicillin) zones of inhibition where observed. EUCAST (2020) zone diameter breakpoint for ampicillin and *Enterobacterales* reports resistance at <14 mm, indicating a change in clinical susceptibility; the corresponding data for cephalothin are not available. For SaCHG, there was no zone of inhibition around oxacillin discs compared to 22.28 mm for the parent strain (EUCAST breakpoint data not available) and for PaGTA and PaBZK there was no zone of inhibition around sulphatriad discs compared to 15.11 mm for the parent strain (EUCAST breakpoint data not available).

**TABLE 2:** Antibiotic susceptibility profiles of pre‐ and post‐primed bacteria strains

Antibiotics (μg)	Zone of inhibition (mm) (SE)						
	EcATCC	EcH2O2	SaATCC	SaCHG	PaATCC	PaGTA	PaBZK
Ampicillin (10)	18.88^a^ (1.50)	NZ^a^	31.90 (0.05)	31.81 (0.14)	NZ	NZ	NZ
Cephalothin (5)	10.43^a^ (0.24)	NZ^a^	31.67 (0.21)	31.84 (0.16)	NZ	NZ	NZ
Colistin (25)	14.41 (0.24)	14.37 (0.23)	NZ	NZ	15.92 (0.59)	15.87 (0.59)	13.86 (0.39)
Gentamicin (10)	21.67 (0.22)	21.90 (0.46)	22.82 (0.084)	22.76 (0.12)	15.96 (0.82)	15.62 (0.58)	13.23 (3.29)
Streptomycin (10)	18.18 (0.94)	17.98 (0.83)	19.11 (0.06)	18.78 (0.21)	10.00 (0.18)	9.75 (0.37)	7.20 (3.60)
Sulphatriad (200)	31.26 (0.62)	31.20 (0.56)	26.13 (0.85)	26.14 (0.83)	15.11^a^ (0.58)	NZ^a^	NZ^a^
Tetracycline (25)	21.99 (1.27)	21.93 (1.27)	23.16 (0.37)	23.09 (0.37)	8.779 (0.05)	8.78 (0.07)	10.00 (0.34)
Cotrimoxazole (25)	27.46 (1.02)	28.11 (0.77)	20.35 (0.19)	20.57 (0.13)	NZ	NZ	NZ
Chloramphenicol (25)	20.42 (0.33)	21.37 (0.33)	20.03 (0.21)	19.97 (0.22)	NZ	NZ	NZ
Erythromycin (5)	NZ	NZ	17.12 (0.42)	17.08 (0.38)	NZ	NZ	NZ
Fusidic Acid (10)	NZ	NZ	26.71 (0.12)	26.50 (0.22)	NZ	NZ	NZ
Oxacillin (5)	NZ	NZ	22.28^a^ (0.44)	NZ^a^	NZ	NZ	NZ
Novabiocin (5)	NZ	NZ	22.39 (0.07)	22.35 (0.02)	NZ	NZ	NZ
Penicillin G 1 unit	NZ	NZ	21.26 (0.24)	21.23 (0.22)	NZ	NZ	NZ
Streptomycin (10)	18.55 (0.99)	19.04 (1.21)	16.13 (0.67)	16.09 (0.61)	8.96 (1.26)	8.96 (1.25)	10.87 (0.34)
Tetracycline (25)	20.77 (0.32)	20.94 (0.15)	21.06 (0.26)	20.94 (0.21)	NZ	NZ	7.82 (0.27)

*Note*: NZ, no zone (6 mm disc diameter). Standard error shown in parentheses (*n* = 3). EcH_2_O_2,_ H_2_O_2_ primed *E. coli*; SaCHG, CHG primed *S. aureus*; PaGTA, GTA primed *P. aeruginosa;* and PaBZK, BZK primed *P. aeruginosa*.

^a^ Changes in zones of inhibition where observed compared to parent strain.

To investigate the resistance more closely, the MIC of selected antibiotics against biocide primed strains of bacteria was determined (summarized in Table 3); the strain and antibiotic combinations tested were based on the results obtained from antibiotic disc diffusion tests. Due to the increased resistance of EcH_2_O_2_ to cephalothin (a first‐generation cephalosporin), both cefoxitin and ceftriaxone, second‐ and third‐generation cephalosporins respectively, were included in the MIC testing to see whether the cross‐resistance extended to newer antibiotics. There was an eightfold increased MIC for EcH_2_O_2_ to cephalothin from 4 mg l^−1^ for the parent strain to 32 mg l^−1^ for EcH_2_O_2_, a fourfold increase in MIC for EcH_2_O_2_ to cefoxitin to 16 mg l^−1^ compared to 4 mg l^−1^ in the parent strain and a twofold increase to ceftriaxone (parent 0.0625 mg l^−1^ and primed strain 0.125 mg l^−1^) and ampicillin (parent 2 mg l^−1^ and primed 4 mg l^−1^). The MIC breakpoint (EUCAST, 2020) for *Enterobacterales* reports resistance at >2 mg l^−1^ with ceftriaxone indicating no change in clinical susceptibility, data for cephalothin and cefoxitin are not available. A fourfold increased MIC for oxacillin was observed in SaCHG (2 mg l^−1^ compared to parent strain *S. aureus* 0.5 mg l^−1^; breakpoint data not available). In PaGTA, an eightfold increased MIC to ciprofloxacin was observed (1 mg l^−1^ compared to 0.125 mg l^−1^ for the parent *P. aeruginosa* strain) and a fourfold increased MIC to sulphatriad was also recorded. The MIC breakpoint (EUCAST, 2020) for *Pseudomonas* spp. and ciprofloxacin reports resistance at >0.5 mg l^−1^ indicating clinically significant resistance in PaGTA (data for sulphatriad are not available).

**TABLE 3:** MICs of selected antibiotics against parent and primed strains of bacteria

Strains	MIC (mg l^−1^)						
	Cephalothin	Cefoxitin	Ceftriaxone	Ampicillin	Oxacillin	Sulphatriad	Ciprofloxacin
*E. coli*	4	4	0.0625	2	—	—	—
EcH_2_O_2_	32^a^	16^a^	0.125^a^	4^a^	—	—	—
*S. aureus*	—	—	—	—	0.5	—	—
SaCHG	—	—	—	—	2^a^	—	—
*P. aeruginosa*	—	—	—	—	—	256	0.125
PaGTA	—	—	—	—	—	1024^a^	1^a^

*Note*: EcH_2_O_2,_ H_2_O_2_ primed *E. coli*; SaCHG, CHG primed *S. aureus*; PaGTA, GTA primed *P. aeruginosa*. Standard error shown in parentheses (*n* = 3).

^a^ Increase in MIC compared to parent strain.

### The effect of efflux pump inhibitors

The MICs of cephalothin and cefoxitin against EcH_2_O_2_ were reduced in the presence of the efflux pump inhibitor TZ (from 32 to 8 mg l^−1^ for cephalothin and from 16 to 8 mg l^−1^ for cefoxitin; Table 4). The MIC of oxacillin against SaCHG was reduced from 2 to 0.25 mg l^−1^ in the presence of TZ and to 0.5 mg l^−1^ in the presence of CPZ (Table 5). The effect of TZ on the MIC of ciprofloxacin and sulphatriad against PaGTA could not be determined due to the turbidity, caused by insolubility, observed when 250 mg l^−1^ of TZ (a concentration that does not inhibit growth) was combined with either ciprofloxacin or sulphatriad. To further determine the effect of TZ in the presence of either antibiotics, contents of the previously observed turbid wells were spread onto agar and further incubated at 37°C for 24 h. Reduced growth on agar of PaGTA was observed from wells containing ciprofloxacin + TZ and sulphatriad + TZ, compared to wells with only ciprofloxacin, TZ or sulphatriad (data not shown). The same observation was seen in the case of PaBZK, where no growth on agar was observed from wells containing 128 mg l^−1^ BZK + TZ, compared to significant growth from wells with only BZK or TZ (data not shown).

**TABLE 4:** The effect of TZ on the tolerance of cephalothin and cefoxitin in EcH_2_O_2_

Strain	MIC (mg l^−1^)				
	TZ	Cephalothin	+ TZ	Cefoxitin	+ TZ
EcH_2_O_2_	128	32	8^a^	16	8^b^

*Note*: EcH_2_O_2_, hydrogen peroxide primed *E. coli*. TZ, thioridazine.

Reduced MIC for ^a^Cephalothin and ^b^cefoxitin was observed in the presence of 40 mg l^−1^ thioridazine.

**TABLE 5:** The effects of TZ and CPZ on oxacillin in SaCHG

Strain	MIC (mg l^−1^), mode *n* = 3				
	TZ	CPZ	Ox	+ ½ TZ	+ ½ CPZ
SaCHG	31.25	64	2	0.25	0.5

*Note*: Reduced MIC to oxacillin was observed in the presence of TZ and CPZ. SaCHG, CHG primed *S. aureus*; TZ, thioridazine; CPZ, chlorpromazine. Standard error shown in parentheses (*n* = 3).

### Confirmation of strain identity

16S rRNA sequencing confirmed all parent (wild type) and biocide primed strains used/generated in this study were at least 99.9% identical to the type strains; *E. coli* ATCC 8739, *S. aureus* ATCC 6538 and *P. aeruginosa* ATCC 15442 (data not shown).

### Fitness of primed strains

**FIGURE 1:**
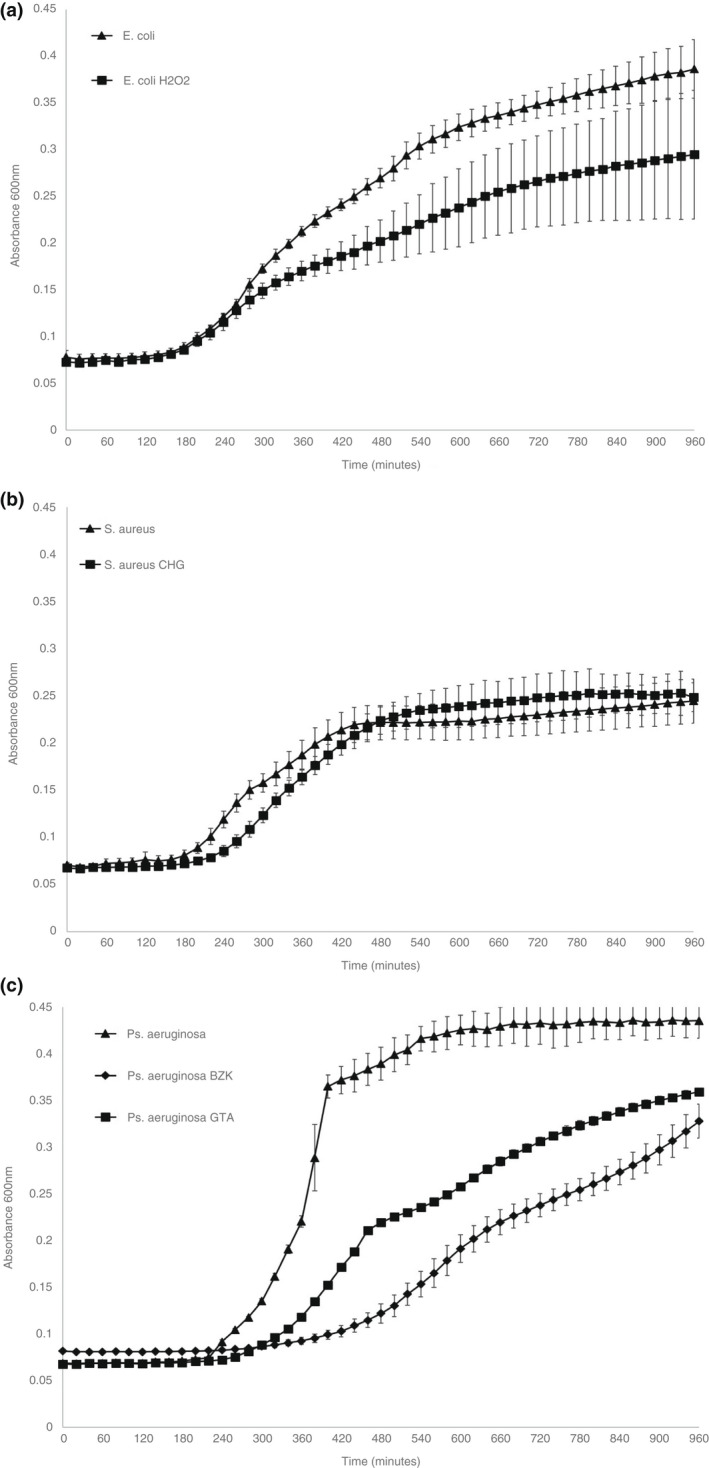
Growth curves of *E. coli* (a), *S. aureus* (b) and *Ps. aeruginosa* (c) primed and parent strains in antibiotic‐free Mueller Hinton broth, absorbance at 600 nm recorded for 16 h (*n* = 3, error bars denote standard deviation)

Results of 16‐h growth curves of parent and primed strains are shown in Figure 1. There was no difference in the length of the lag phase between the *E. coli* parent strain and primed isolate, but growth was decreased from 4 h onwards (Figure 1a). For *S. aureus*, the CHG primed isolate had an extended lag phase compared to the parent strain, but overall growth was the same from 6 h onwards (Figure 1b). Interestingly, both the *P. aeruginosa* BZK and GTA isolates had an extended lag phase and decreased overall growth when compared to the parent strain (Figure 1c). This effect was more pronounced with the BZK isolate.

## DISCUSSION

The goal of the present study was to test whether biocide priming has an effect on bacterial resistance to antibiotics. Low concentrations of H_2_O_2_ have previously been shown to promote tolerance to the biocide (Bogomolnaya et al., 2013; Imlay & Linn, 1987) as was initially seen in this study with *E. coli*. In contrast to previous studies, here the increased tolerance to H_2_O_2_ was short lived. Stable cross‐resistance of EcH_2_O_2_ to cephalothin, cefoxitin, ceftriaxone and ampicillin antibiotics was observed, similar to the findings of Pereira et al. (2021). Previous studies show H_2_O_2_ activates the SoxRS and the OxyR regulons (Aslund et al., 1999; Manchado et al., 2000) both of which control the expression of over 100 oxidative stress response genes (Blanchard et al., 2007, 2012; Demple, 1991), including *acrAB*, which encode a multidrug efflux pump (Dukan et al., 1996; Storz & Imlay, 1999). Although this might explain the cross‐tolerance to antibiotics after H_2_O_2_ priming observed here, further work is required to see if either the SoxRS or OxyR regulons are activated in the present study, by comparing the expression levels of genes involved in both parent and primed strains. This could help to establish the mechanism(s) behind the differences seen between this study and others. Specifically, the ability of biocides to prime for antibiotic resistance without the usual observation of stable biocidal tolerance. Exposure of *S. aureus* to CHG did not reduce its susceptibility to the biocide, contrasting the findings of Hardy et al. where prolonged exposure of *S*. *aureus* isolates to CHG led to reduced susceptibility (Hardy et al., 2017). Here, cross‐resistance to oxacillin was observed with no corresponding tolerance to the biocide.

The results from the present study show an increased MIC to BZK in PaBZK in comparison to the parent *P. aeruginosa* strain. Activation of multidrug efflux systems such as the RND‐type MexCD‐OprJ was previously linked to the use of sub‐inhibitory concentrations of membrane damaging biocides including BZK and CHG (Fraud et al., 2008; Morita et al., 2003). Previous adaptation of *P. aeruginosa* to sub‐inhibitory concentrations of BZK was attributed to increased efflux (Loughlin et al., 2002; McCay et al., 2010). In their work, McCay et al. (2010) observed cross‐resistance with ciprofloxacin in BZK primed *P. aeruginosa* grown in continuous culture, in contrast to this study whereby no cross‐resistance to antibiotics, including ciprofloxacin, was seen. This could be attributed to the different methodology used when priming strains as here a serial batch method, gradient plating, was used in comparison to the continuous culture method employed by McCay et al. (2010). Our findings are consistent with those of Loughlin et al., where cross‐resistance was observed with BZK and other quaternary ammonium compounds after serial batch culture (Loughlin et al., 2002). The varied outcome in these studies shows how different growth conditions may influence adaptation and selection of bacteria to antimicrobials.

There are several reports of bacterial resistance to GTA (Kampf et al., 2013; Kirschke et al., 2003; Simoes et al., 2006; Simões et al., 2011; Svetlikova et al., 2009; Tschudin‐Sutter et al., 2011), which was not observed in this study. Cross‐resistance after priming with GTA to two unrelated antibiotics, ciprofloxacin and sulphatriad, was however observed. A study using *Pseudomonas fluorescens* biofilms showed that exposure of the bacteria to GTA significantly induced expression of two genes encoding multidrug efflux pumps, PFLU2929 and PFLU3876, which appear to be orthologs of OprN and PA5159 in *P. aeruginosa* (Vikram et al., 2015). The cross‐resistance observed with unrelated antibiotics in this study might suggest efflux is involved, as seen previously (Ferreira et al., 2011; Maseda et al., 2009; Poole, 2002; Sanchez et al., 2005).

Phenothiazines such as TZ and CPZ have been shown to potentiate the effect of antimicrobials against bacteria (Viveiros & Amaral, 2001; Wainwright et al., 1998), eliminate antibiotic resistant plasmids (Evdokimova et al., 1997; Radhakrishnan et al., 1999) and inhibit bacterial efflux pumps (Costa et al., 2013; Machado et al., 2018; Ordway et al., 2003; Viveiros & Amaral, 2001). The most well‐studied efflux pump inhibitor is CPZ, but both CPZ and TZ have the same antimicrobial properties against efflux and phagocytosed bacteria (Machado et al., 2018; Ordway, Viveiros, Leandro, Arroz, & Amaral, 2002; Ordway, Viveiros, Leandro, Arroz, Molnar, et al., 2002). Here we tested the effect of TZ at sub‐inhibitory concentration on EcH_2_O_2_ in the presence of varying concentrations of cephalothin and cefoxitin, to which the primed strain had previously shown cross‐resistance. TZ greatly increased the susceptibility of EcH_2_O_2_ to cephalothin and cefoxitin, suggesting that efflux mechanisms may be involved in the observed cross‐resistance. Our results corroborate the findings of Amaral et al. where CPZ reduced the MIC of ceftazidime and ceftriaxone against *E. coli* from 1.0 to 0.08 mg l^−1^ and 0.07 mg l^−1^, respectively (Amaral et al., 1992). These findings suggest that increased efflux might be a contributory factor in the cross‐resistance observed with EcH_2_O_2_.

When the effect of both TZ and CPZ on MIC levels of oxacillin on SaCHG was evaluated, the results revealed a significant reduction of MIC in the presence of CPZ and TZ. These results suggest that efflux mechanisms may be contributing to the cross‐resistance observed. Furthermore, this agrees with the findings of Kristiansen et al. (2006) and Costa et al. (2013).

Although efflux contributes highly to antimicrobial resistance in *P. aeruginosa*, reduced influx/impermeability has also been shown to contribute (Li et al., 2000), for example mutation in or loss of the transmembrane porin OprD is significant in resistance to carbapenems (Bradford et al., 1999). Thus, a combination of increased efflux and decreased influx may contribute to antimicrobial resistance in *P. aeruginosa* resistance, even though it was not fully demonstrated with the methods used here.

The increased susceptibility seen with some biocide primed strains to antibiotics in the presence of EPIs suggests efflux as a contributory mechanism to the cross‐resistance observed in this study. The next step will be to employ the use of q qPCR to compare gene expression in parent and primed strains of bacteria.

The growth curve results comparing both parent and primed strains indicated that the resistance phenotype did have an impact on overall growth in the case of *E. coli*, the length of the lag phase with *S. aureus* and both overall growth and lag phase with *P. aeruginosa*. This suggests that although there may be potential fitness cost associated with these phenotypic adaptations in terms of initial or overall speed of replication, this is not impacting on the conservation of the resistance after repeated subculture under laboratory conditions. This agrees with other findings, where generally most mutations had an impact on fitness (Melnyk et al., 2015). It is not unexpected to observe this with biocide induced cross‐resistance, as biocides generally have broader modes of action in terms of cellular targets.

Results from this study clearly demonstrate that continuous exposure to sub‐inhibitory concentrations of biocides far below the recommended in‐use concentration can, under laboratory conditions, prime bacteria to become resistant to antibiotics even in the absence of increased tolerance to the biocides. This raises the important question of whether this phenomenon is occurring in clinical settings and contributing to dissemination to antimicrobial resistance.

## CONFLICT OF INTEREST

None declared.
